# Novel molecular typing reveals the risk of recurrence in patients with early-stage papillary thyroid cancer

**DOI:** 10.1186/s13044-024-00193-9

**Published:** 2024-04-01

**Authors:** Mingyu Sun, Bingqing Zhao, Tao Chen, Lijun Yao, Xiaoxin Li, Shaojun Hu, Chengling Chen, Xinbao Gao, Chuangang Tang

**Affiliations:** 1 Department of Breast Surgery, Xuzhou Central Hospital, The Affiliated Xuzhou Hospital of Medical College of Southeast University, Xuzhou, 221009 China; 2 Department of Plastic and Reconstructive Surgery, Tianjin Hospital of ITCWM Nankai Hospital, Tianjin, 300100 China; 3 The Xuzhou Clinical College of Xuzhou Medical University, Jiangsu, 221009 China; 4 Department of Oncology, Suzhou Ninth People’s Hospital, Suzhou, 215200 China; 5 Department of Pathology, Xuzhou Central Hospital, The Affiliated Xuzhou Hospital of Medical College of Southeast University, Xuzhou, 221009 China; 6 Department of Surgery for Vascular Thyroid and Hernia, Xuzhou Central Hospital, The Affiliated Xuzhou Hospital of Medical College of Southeast University, Xuzhou, 221009 China

**Keywords:** Thyroid cancer, Recurrence, DNA damage repair, Risk model

## Abstract

**Background:**

Papillary thyroid cancer (PTC) is an indolent disease with a favorable prognosis but characterized by a high recurrence rate. We aimed to improve precise stratification of recurrence risk in PTC patients with early stage using multi-gene signatures.

**Patients and methods:**

The present study was performed using data from The Cancer Genome Atlas (TCGA) and multi-center datasets. Unsupervised consensus clustering was used to obtain the optimal molecular subtypes and least absolute shrinkage and selection operator (LASSO) analysis was performed to identify potential genes for the construction of recurrence signature. Kaplan-Meier survival analysis and the log-rank test was used to detect survival differences. Harrells concordance index (C-index) was used to assess the performance of the DNA damage repair (DDR) recurrence signature.

**Results:**

Through screening 8 candidate gene sets, the entire cohort was successfully stratified into two recurrence-related molecular subtypes based on DDR genes: DDR-high subtype and DDR-low subtype. The recurrence rate of DDR-high subtype was significantly lower than DDR-low subtype [HR = 0.288 (95%CI, 0.084–0.986), *P* = 0.047]. Further, a two-gene DDR recurrence signature was constructed, including PER1 and EME2. The high-risk group showed a significantly worse recurrence-free survival (RFS) than the low-risk group [HR = 10.647 (95%CI, 1.363–83.197), *P* = 0.024]. The multi-center data demonstrated that proportion of patients with low expression of PER1 and EME2 was higher in the recurrence group than those in the non-recurrence group.

**Conclusions:**

These findings could help accurately and reliably identify PTC patients with high risk of recurrence so that they could receive more radical and aggressive treatment strategies and more rigorous surveillance practices.

**Supplementary Information:**

The online version contains supplementary material available at 10.1186/s13044-024-00193-9.

## Background

Thyroid cancer is the most common malignant tumor of the endocrine system, and can be divided pathologically into four major types: papillary thyroid cancer (PTC), follicular thyroid cancer (FTC), medullary thyroid cancer (MTC), and anaplastic thyroid cancer (ATC) [1]. Both PTC and FTC originate from the thyroid follicular cells and carry a favorable survival, collectively referred to as differentiated thyroid cancer (DTC) [2]. In recent decades, the incidence of thyroid cancer has been annually increased primarily due to the increase of the diagnosis of PTC at early stage [3, 4]. The latest American Joint Committee on Cancer (AJCC) staging system for PTC modified the cut-off value of age from 45 to 55, which further increased the proportion of early-stage PTC [5]. According to statistics, PTC make up the vast majority of all thyroid cancers and PTC with stage I accounted for more than 80% of all PTC [6, 7]. The overall 10-year survival rate of PTC is approximately 90% [8], and the10-year survival rate of PTC with stage I is up to 95% [4]. However, the recurrence rate is relatively high, ranging from 10 to 30%, even if patients are in the early stage of PTC [9, 10]. Thewjitcharoen et al. [6] showed that 23.7% of PTC patients with stage I fell into the high risk of recurrence group according to the 2015 American Thyroid Association (ATA) risk classification, indicating that PTC patients with the same survival prognosis also had heterogeneous tumors with different risk of recurrence. Thus, there is a great need to stratify early-stage PTC patients by the risk of recurrence more accurately and reduce their recurrence risk with personalized treatment strategies.

Multiple studies have been dedicated to investigating the risk of recurrence in PTC and have successfully developed risk models. The proto-oncogenes *PBF (PTTG1IP)* and *PTTG1* were linked to recurrence in PTC and the *PBF/PTTG1* signature displayed a notable discriminatory power [11, 12]. A new combinatorial 5-gene risk score classifier incorporated mRNAs and microRNAs, which conducted thorough explorations to achieve a more accurate prediction of recurrence in PTC [13]. Read et al. [14] developed a 13-gene risk score classifier to assess the risk of recurrence in PTC with ablative radioiodine treatment. In their most recent work, they further identified an AP2 gene-related risk score classifier as an effective tool for predicting the recurrence of BRAF-mutant PTC and PTC with ablative radioiodine treatment [15]. However, these studies did not differentiate subgroups of PTC based on clinical staging, which was unfavorable for facilitating appropriate personalized management for PTC patients. Therefore, we aimed to improve precise stratification of recurrence risk in PTC patients with stage I aged < 55 years using multi-gene signatures.

## Patients and methods

### Dataset

Patients diagnosed as thyroid cancer were included from the TCGA-THCA dataset, downloaded from The Cancer Genome Atlas (TCGA, http://tcga-data.nci.nih.gov/tcga/). The exclusion criteria were as follows: (1) diagnosed as FTC, MTC or ATC; (2) 55 years old or older; (3) distant metastasis; (4) unavailable recurrence information. RNA sequences (FPKM) of 191 samples with basic clinical characteristics, including gender, age, pathological T category, pathological N category, ATA recurrence risk stratification, and recurrence status, were downloaded. Gene names were converted using the Ensemble database (http://www.ensembl.org/index.html).

A series of gene sets potentially involved in key pathological mechanisms in PTC were collected from literature, including DNA damage repair (DDR)-related gene set, immune-related gene set, energy metabolism-related gene set, hypoxia-related gene set, ferroptosis-related gene set, lipid metabolism-related gene set, endothelial-mesenchymal transition (EMT)-related gene set, and m6A-related gene set [16–21]. Detailed gene sets were provided in Supplementary Table 1.

### Consistency analysis

Unsupervised consensus clustering was performed using R package ConsensusClusterPlus. 80% of the overall samples were randomly subsampled with 100 iterations. The number of clusters ranged from two to six. The cumulative distribution function (CDF) curves were used to obtain the optimal number of clusters. The relative changes in the area under the CDF curves were also calculated. The heatmap of clustering and gene expression was drawn using R package pheatmap. Genes with standard deviation (SD) less than 0.1 were not shown in the gene expression heatmap. Kaplan-Meier survival analysis and the log-rank test was performed to detect survival differences between two or multiple subgroups using R package survival and survminer.

### Least Absolute Shrinkage and Selection Operator (LASSO) and Receiver Operating Characteristic (ROC)

LASSO regression analysis was performed to identify potential recurrence-associated genes for the construction of DDR-signature. A 10-fold cross-validation scheme was applied. Each gene was assigned a coefficient and the risk score was calculated as follows: Risk score = β1*expression mRNA1 + β2*expression mRNA2 +… + β6*expression mRNA6. More and more coefficients were set gradually to zero, and the top contributing recurrence‐associated genes would be screened. The accuracy of DDR-signature to predict the probable 1‐year, 5‐year and 10‐year recurrence rates was evaluated by the ROC curve and the area under the ROC curve (AUC). LASSO regression analysis and ROC curve analysis were performed using R package glmnet and timeROC, respectively. Harrells concordance index (C-index) was used to assess the performance of the DDR recurrence signature and ATA recurrence risk stratification.

### Immunohistochemistry

PTC patients with recurrence were collected between 2014 and 2016 from multi-center. All the patients from the multicenter datasets were followed up for at least 3 years. Patients with recurrence occurring within 6 months were excluded.

All paraffin-embedded tissues were assembled into tissue chips and immunohistochemistry was performed to detect the protein expression levels of DDR-signature genes (*PER1* and *EME2*). The primary antibodies were Rabbit anti-*PER1* (BOSTER Biological Technology, Wuhan, China; 1:100) and Rabbit anti-*EME2* (Bioss Inc., Beijing, China; 1:100). Secondary antibodies were used at a dilution of 1:10000. The Research Resource Identifier (RRID) numbers of primary and secondary antibodies were provided in Supplementary Table 2. The tissue chips were evaluated by two independent experienced pathological physicians and an additional pathological physician was included in case of inconsistency. Pathological physicians did not have prior knowledge of basic clinical characteristics and recurrence status. The immunohistochemical staining score was calculated by summing the percentage score (proportion of positive stained cells) and the staining intensity score. The percentage score was defined as 0 (0%), 1 (> 0, ≤ 25%), 2 (> 25%, ≤ 50%), 3 (> 50%, ≤ 75%), and 4 (> 75%, ≤ 100%). The staining intensity score was defined as 0 (negative), 1 (weak), 2 (moderate), and 3 (strong). The entire cohort was ranked according to immunohistochemistry scores from low to high. The top 50% patients were considered as high expression. The bottom 50% patients were considered as low expression. Written consent was obtained from all participants. Samples were collected and disposed of in accordance with WHO guidelines. The study was approved by the Ethics Committee of Suzhou Ninth People’s Hospital.

### Statistical analysis

Statistical analysis was performed with R software version 3.6.2 (The R Foundation). Hazard ratio (HR) and its 95% confidence interval (95% CI) were calculated by the log-rank test and univariate Cox regression analysis. Multivariate Cox regression analysis was used to determine the independent predictors. P value less than 0.05 was considered statistically significant.

## Results

### Basic clinical characteristics

A total of 191 PTC patients with stage I aged < 55 years were included from the TCGA database (Table 1). The age ranged from 15 to 54 years with the median age of 39 years. Females were responsible for the majority (76.4%, 146/191). The entire cohort was almost equally distributed across three pathological T categories. The number of PTC patients without LN metastasis was the same as those with LN metastasis. The follow-up period ranged from 0 to 14.1 years with the median follow-up period of 2.5 years. 11 patients had recurrences over the course of follow-up.

**Table 1:** Basic clinical characteristics of PTC patients with stage I aged < 55 years

Characteristics	PTC (*n* = 191)
Age (median)	39
Gender	
Female	146 (76.4%)
Male	45 (23.6%)
Pathological T category	
T1	61 (31.9%)
T2	64 (33.5%)
T3	63 (33.0%)
T4	2 (1.1%)
TX	1 (0.5%)
Pathological N category	
N0	92 (48.2%)
N1	92 (48.2%)
NX	7 (3.6%)
Pathological M category	
M0	191 (100%)
ATA recurrence risk stratification *	
Low	61 (31.9%)
Intermediate	92 (48.1%)
High	2 (1%)
Recurrence	
Yes	11 (5.8%)
No	180 (94.2%)

*36 cases missing

### Molecular typing based on DDR genes

**Fig. 1:**
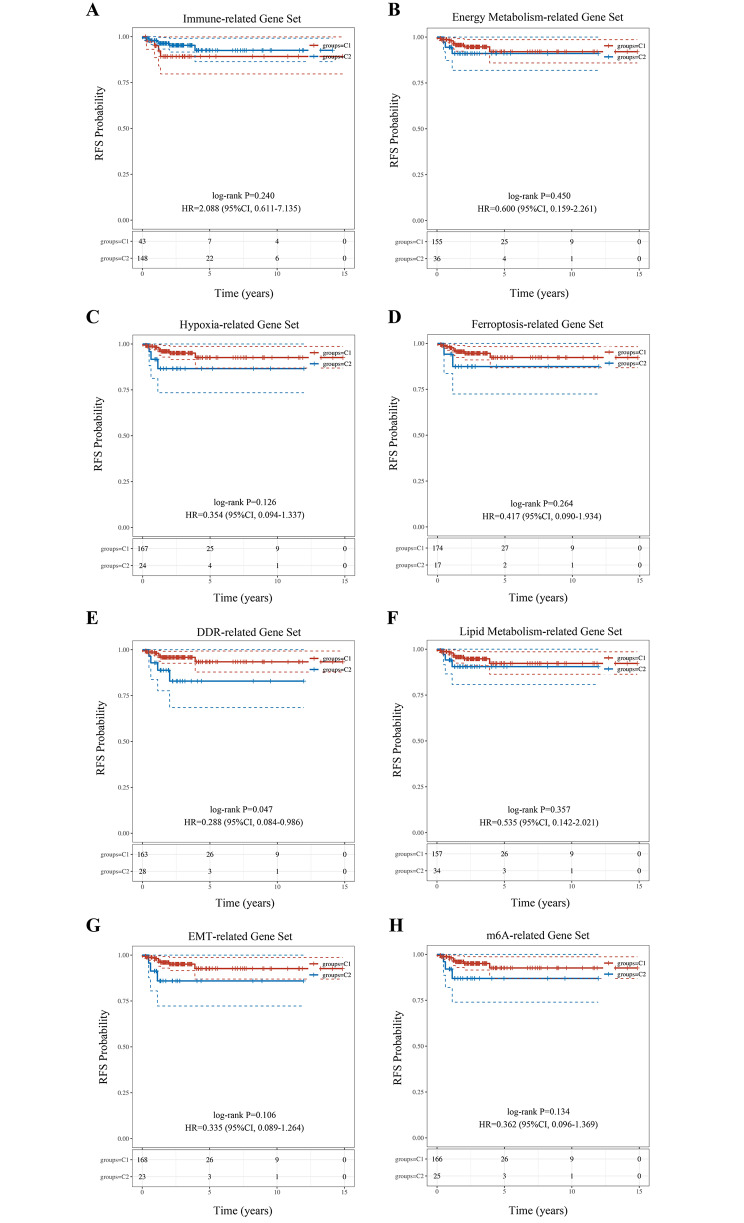
Survival curves categorized by 8 different gene sets. **A** Survival curve categorized by immune-related gene set. **B** Survival curve categorized by energy metabolism-related gene set. **C** Survival curve categorized by hypoxia-related gene set. **D** Survival curve categorized by ferroptosis-related gene set. **E** Survival curve categorized by DDR-related gene set. **F** Survival curve categorized by lipid metabolism-related gene set. **G** Survival curve categorized by EMT-related gene set. **H** Survival curve categorized by m6A-related gene set

**Fig. 2:**
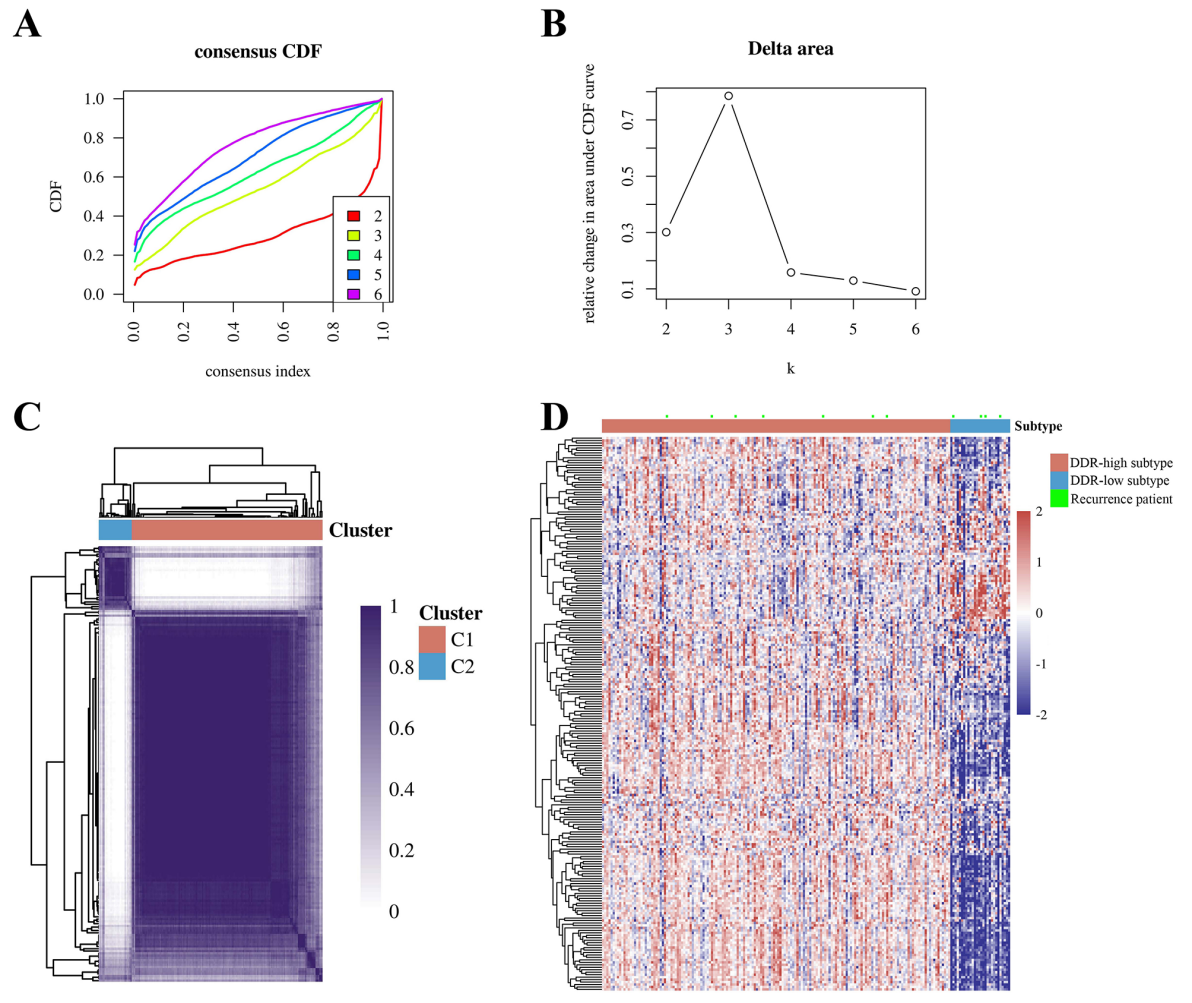
Two molecular subtypes categorized by DDR-related gene set. **A** Consensus clustering model with CDF for k = 2–6. **B** Relative change in the area under the CDF curve k = 2–6. **C** Entire cohort was divided into two distinct clusters (k = 2). **D** Heatmap of gene expression in two distinct clusters (cluster 1 was named DDR-high subtype and cluster 2 was named DDR-low subtype)

Using the above 8 gene sets, we performed unsupervised consensus clustering to identify distinct subgroups of early PTC with different risks of recurrence. No statistically significant different subgroups were identified with the exception of DDR-based subgroups [HR = 0.288 (95%CI, 0.084–0.986), *p* = 0.047] (Fig. 1). This suggested that DDR might play a crucial role in tumor recurrence of early-stage PTC. According to the CDF values and the R package SigClust evaluation, k = 2 was determined as the best clustering solution (Fig. 2A and B). The consensus clustering matrix showed that k = 2 could clearly stratify samples into two apparently different clusters (Fig. 2C). Cluster 1 (C1) comprised 163 samples and Cluster 2 (C2) comprised 28 samples. As shown in the gene expression heatmap of DDR (Fig. 2D), the majority of DDR genes were highly expressed in C1 and associated with a relatively low risk of recurrence (named DDR-high subtype). Conversely, the majority of DDR genes were down-regulated in C2 and associated with a relatively high risk of recurrence (named DDR-low subtype).

### Construction of DDR recurrence signature

**Fig. 3:**
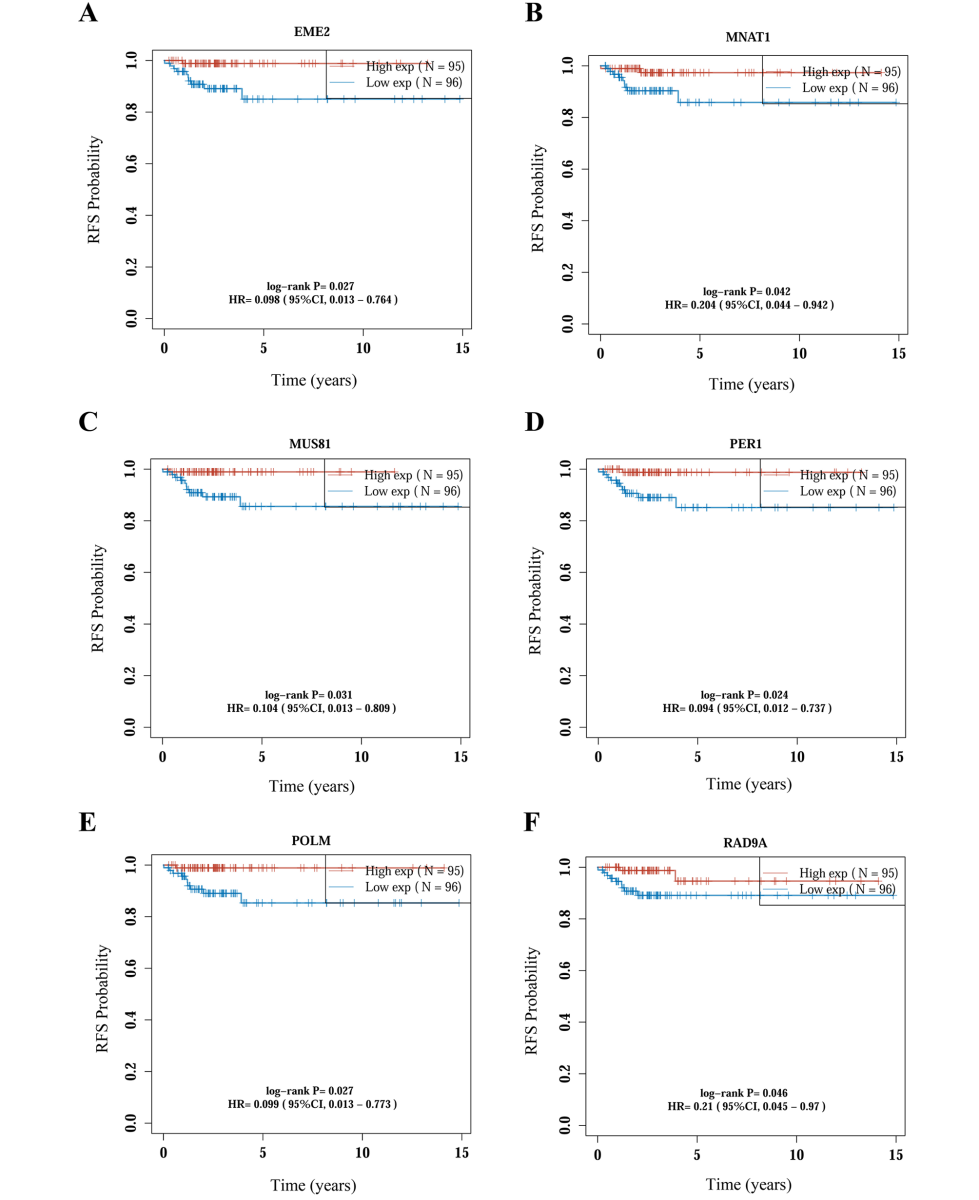
The RFS curves of 6 DDR genes with prognostic significance in early-stage PTC. **A** The RFS curve according to the expression of EME2. **B** The RFS curve according to the expression of MNAT1. **C** The RFS curve according to the expression of MUS81. **D** The RFS curve according to the expression of PER1. **E** The RFS curve according to the expression of POLM. **F** The RFS curve according to the expression of RAD9A

According to the expression level of each DDR gene, the patient cohort was correspondingly classified into high-expression and low-expression groups. The differences in RFS between the two groups were evaluated using Kaplan–Meier survival analysis with log-rank test. The results revealed 6 DDR genes with prognostic significance in relation to patient RFS, including *EME2, MNAT1, MUS81, PER1, POLM*, and *RAD9A* (Fig. 3, *p* < 0.05). Interestingly, the lower expression levels of these DDR genes were associated with a higher probability of experiencing recurrence in early-stage PTC. The detailed information of all DDR genes with HRs and 95%CI was provided in Supplementary Table 3. To be clear, the *p*-values shown were unadjusted values.

**Fig. 4:**
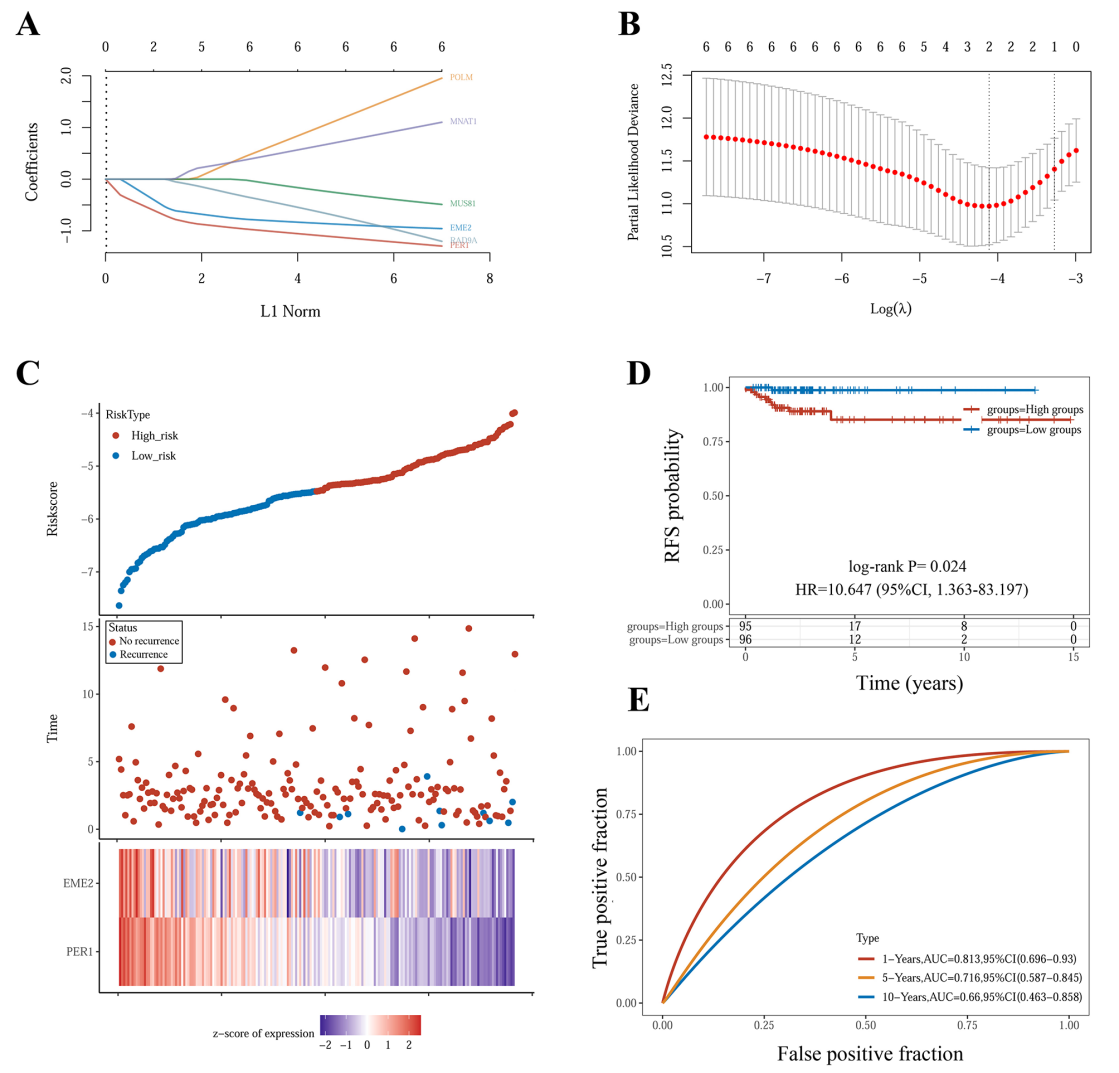
Construction of DDR recurrence signature. **A** Lasso regression analysis. **B** Partial likelihood deviance for different genes and numbers. **C** Patient distribution according to risk score from small to large. **D** The RFS curve according to different risk groups. **E** The AUC of DDR recurrence signature for the prediction of 1-year, 5-year, and 10-year RFS.

Based on the above 6 DDR genes in relation to RFS, a LASSO regression model was constructed to develop a DDR recurrence signature. The minimal λ was 0.0164, and two genes (*PER1* and *EME2*) were screened for the final signature (Fig. 4A and B). The recurrence risk score of each sample was obtained using the following formula: Risk score = (-0.664)*PER1 + (-0.483)*EME2. Multivariate Cox regression analysis showed that the risk score could be taken as an independent prognostic factor for RFS in early-stage PTC patients (Table 2). The patient distribution was shown in Fig. 4C according to risk score from small to large. The PTC patients with recurrence were largely distributed in the region with higher risk score. The mRNA expression levels of *PER1* and *EME2* decreased gradually with the elevation of recurrence risk score. According to the median risk score, the entire cohort was divided into high-risk group and low-risk group. The high-risk group showed a significantly worse RFS than the low-risk group [HR = 10.647 (95%CI, 1.363–83.197), *p* = 0.024] (Fig. 4D). The AUC of the DDR recurrence signature for the prediction of 1-year RFS was 0.813 (95%CI, 0.696–0.930), indicating that the signature could accurately reflect the recurrence of early-stage PTC (Fig. 4E). Due to lower recurrence rate, however, the signature did not exhibit as highly predictive power for 10-year RFS as 1-year RFS.

**Table 2:** Multivariate Cox analysis for RFS of PTC patients with stage I aged < 55 years

Characteristics	HR (95%CI)	*P* value
Age	1.034(0.963–1.110)	0.359
Gender		
Male	Reference	
Female	0.951(0.191–4.741)	0.951
Pathological T category		
T1	Reference	
T2	2.606(0.502–13.519)	0.254
T3	1.846(0.313–10.876)	0.498
Pathological N category		
N0	Reference	
N1	1.182(0.317–4.402)	0.803
Risk score	5.286(1.780-15.699)	0.003

### Multicenter validation

**Fig. 5:**
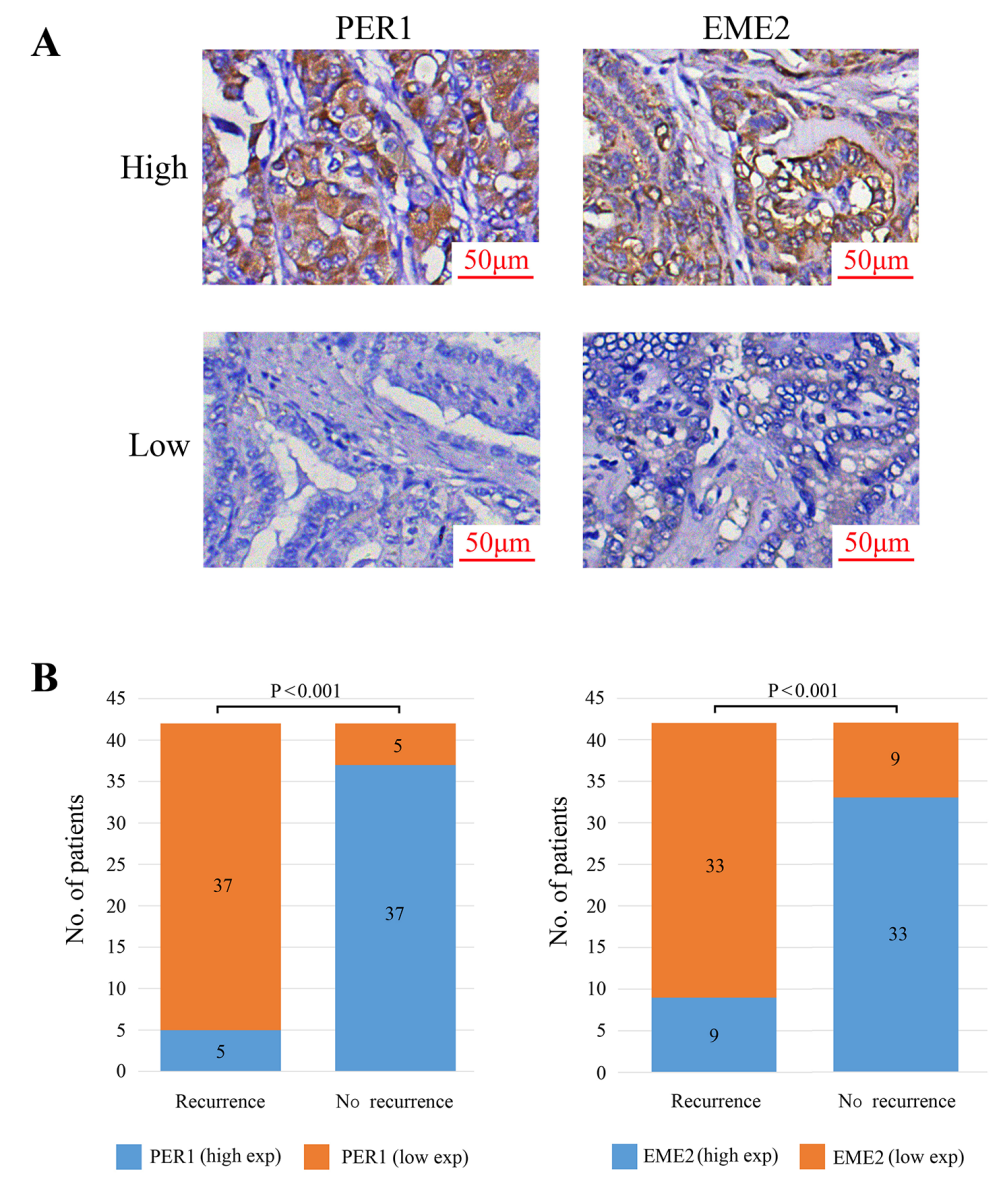
Immunohistochemical analysis of clinical samples from multi-center datasets. **A** Immunohistochemical staining for PER1 and EME2. **B** Statistical analysis for PER1 and EME2 protein staining

42 early-stage PTC patients with recurrence were collected in the multicenter datasets between 2014 and 2016. Over the same period, a total of 629 early-stage PTC patients without recurrence were recorded. To eliminate intergroup selection bias, 1:1 propensity score matching (PSM) was performed by adjusting baseline characteristics, including age, gender, pathological T category, and pathological N category. 42 pairs of patients (42 cases in the recurrence group and 42 cases in the non-recurrence group) were finally included in the further validation using immunohistochemical analysis (Fig. 5A, Supplementary Fig. 1, Supplementary Table 4). In the recurrence group, 88.1% of patients had low expression of *PER1*, which was significantly higher than that in the non-recurrence group (Fig. 5B, *p* < 0.001). Similarly, the proportion of patients with low expression of *EME2* in the recurrence group was also significantly higher than that in the non-recurrence group (78.6% vs. 21.4%, Fig. 5B, *p* < 0.001).

### Comprehensive assessment of DDR recurrence signature

**Fig. 6:**
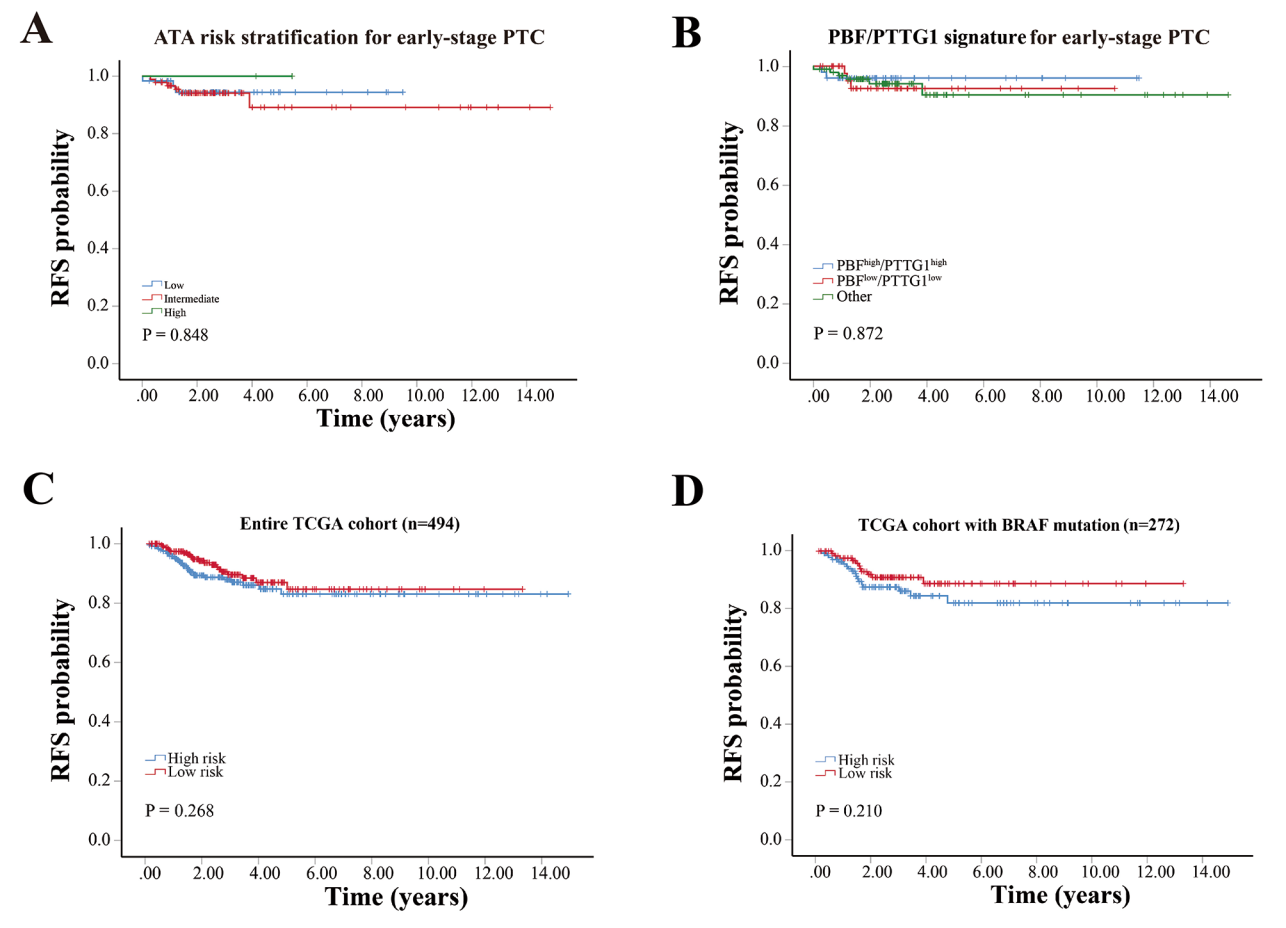
Comprehensive assessment of DDR Recurrence Signature. **A** The RFS curve for early-stage PTC according to the ATA risk stratification. **B** The RFS curve for early-stage PTC according to the PBF/PTTG1 signature. **C** The application of the DDR signature in the entire TCGA cohort. **D** The application of the DDR signature in the TCGA cohort with BRAF mutation

We compared the discriminatory abilities of the DDR recurrence signature and ATA recurrence risk stratification. The C-index for the DDR recurrence signature was 0.701 (95% CI = 0.561–0.841), whereas for the ATA recurrence risk stratification it was 0.509 (95% CI = 0.378–0.640) (Fig. 6A). These indicated the DDR recurrence signature outperformed the ATA recurrence risk stratification in the prediction of recurrence risk for early-stage PTC.

Next, the performance of the DDR recurrence signature and published *PBF/PTTG1* signature were compared [11]. Although the *PBF/PTTG1* combined signature displayed a notable discriminatory power for all-stage PTC, its performance in distinguishing early-stage PTC remained to be evaluated. Regretfully, the *PBF/PTTG1* signature was not a reliable predictor for recurrence risk in early-stage PTC (Fig. 6B).

Finally, we further investigated the effectiveness of the DDR recurrence signature in predicting recurrence for all-stage PTC using the entire TCGA cohort. Unfortunately, the DDR recurrence signature did not exhibit strong predictive capability for recurrence in all-stage PTC (Fig. 6C). Even for BRAF-mutated PTC with a higher recurrence rate, the predictive performance of the DDR recurrence signature was not sufficiently satisfactory either (Fig. 6D), suggesting that the DDR recurrence signature may function as a recurrence risk model only for early-stage PTC.

## Discussion

PTC is generally an indolent disease with extremely good survival outcomes and concern arises as to whether some PTC patients are over-treated, particularly PTC patients with early stage. It was for the reason that the ATA guidelines recommended lobectomy as an appropriate treatment method for solitary intrathyroidal PTC without lymph node or distant metastases (low risk of recurrence patients according to the ATA guidelines). However, there is currently no strong supporting clinical evidence for the recommendation. Huang et al. [22] classified the low risk of recurrence patients based on the BRAF V600E status, with a view to finding a subgroup of patients with high risk of recurrence. The study also has several shortcomings that should be addressed. First, the prevalence of BRAF V600E mutation in early-stage PTC was significantly lower than the average of all PTC patients [22–24]. Second, multi-gene signature might provide more accurate stratification of risk. Using the TCGA database and multi-center datasets, we proposed a novel molecular subtype of PTC patients with stage I aged < 55 years based on DDR, which were associated with disparate risks of recurrence. Further, a two-gene DDR recurrence signature (*PER1* and *EME2*) was constructed, which could accurately and reliably predict the risk of recurrence. These findings indicated that more radical and aggressive treatment strategies and more rigorous surveillance practices might be appropriate for early-stage PTC patients with low expression of *PER1* or *EME2*.

*PER1* (period 1), one of circadian clock genes, was expressed at low levels in multiple cancers and reported to be involved in tumor progression and prognosis via multiple biological pathways, in particular via DDR pathways and cell apoptosis [25, 26]. Jiang et al. [27] developed a ten-gene signature including *PER1*, which could effectively predict the disease-free survival (DFS) of patients with hepatocellular cancer. High expression of *PER1* was also found to be associated with metastasis-free survival (MFS) of patients with breast cancer [28], suggesting an important role of *PER1* in the early stages of tumor progression. However, there are no reports about the effect of *PER1* on the prognosis of PTC patients at present. Our study found that the low expression of *PER1* was significantly associated with high risk of recurrence in early-stage PTC, which was in good agreement with previous studies for other cancers [29]. *PER1* promoted DDR-induced apoptosis via *p53/Myc* pathway in different tumor cells, while *p53* repressed the transcription of *PER1* [30, 31]. These indicated that *PER1* might decelerate the relapse process of early-stage PTC by interacting with *p53*.

*EME2*, essential meiotic structure-specific endonuclease subunit 2, is localized on chromosome 16p13.3. *EME2*, *EME1*, and *MUS81* belong to the *XPF/MUS81* family of structure-selective endonucleases [32]. It has previously been suggested that *MUS81* participated in various cellular functions only through *MUS81-EME1* complex [33]. Recent studies indicated that *MUS81-EME2* complex was involved in the process of fork cleavage and restart during DNA replication [34]. Our study also showed that both *EME2* and *MUS81* were significant predictors of early-stage PTC recurrence, suggesting *EME2* and *MUS81* might act in the form of complex in PTC. Kiminori et al. [35] showed that depletion of *MUS81-EME2* complex and *Polη* (a Y-family translesion synthesis polymerase) would synergistically enhanced *Myc*-induced replication stress, contributing to carcinogenesis. Additionally, simultaneous deficiency of *MUS81* and *p53* greatly increased the likelihood of sarcoma development in mice. These suggested that *MUS81-EME2* complex, *PER1*, and *p53/Myc* pathway may collectively contribute to the molecular mechanisms involved in the recurrence of early-stage PTC. It has been shown that the well-known proto-oncogenes *PBF* and *PTTG1* not only modulated the expression of DDR genes, but also exerted effects on the activity of *p53* [11]. Therefore, we speculate that *PBF/PTTG1* may be upstream regulatory genes for complex molecular networks including *MUS81-EME2*, *PER1*, and *p53/Myc* in early-stage PTC. Certainly, further experiments are needed to confirm this deduction.

Although the other genes related to RFS were not included in the DDR signature, they were reported to have an impact on the tumor progression in malignancies. The single nucleotide polymorphism (SNP) of *MNAT1* (rs12888332) was a significant predictor of recurrence of head and neck cancer [36]. Long et al. [37] established a RFS nomogram model of prostate cancer including *POLM*, which could accurately discriminated patients at high and low risk of recurrence. Eva et al. [38] found that reduced expression of *RAD9A* was associated with high risk of developing secondary cancer in children with a history of cancer.

There were a few limitations in the present study. First, all the data from TCGA and multi-center were retrospective. Prospective, multi-center and large-sample clinical trials are needed to provide guidance for individualized precision therapy. Second, as PTC patients with stage I aged < 55 years usually have an excellent prognosis, the duration of follow-up seemed not sufficiently long. Third, the study did not address animal or in vitro cellular experiments, leading to insufficient mining of molecular mechanisms behind recurrence in early-stage PTC.

## Conclusions

In sum, we proposed a novel molecular subtype of PTC patients with stage I aged < 55 years based on DDR, and further constructed a two-gene signature (*PER1* and *EME2*) for early-stage PTC recurrence prediction. These findings could help accurately and reliably identify PTC patients with high risk of recurrence so that they could receive more radical and aggressive treatment strategies and more rigorous surveillance practices.

### Electronic supplementary material

Below is the link to the electronic supplementary material.

Supplementary Fig.1. Representative images of IHC

Supplementary Table 1. Detailed list of 8 gene sets

Supplementary Table 2. List of antibodies with RRID numbers

Supplementary Table 3. All DDR genes with HRs and 95%CI

Supplementary Table 4. Detailed data of multicenter cohort

## Data Availability

The data that support the findings of this study are available from the corresponding authors upon reasonable request. The training dataset was obtained from the TCGA data portal (https://portal.gdc.cancer.gov/).

## Abbreviations

PTC: Papillary thyroid cancer
FTC: Follicular thyroid cancer
MTC: Medullary thyroid cancer
ATC: Anaplastic thyroid cancer
AJCC: American Joint Committee on Cancer
ATA: American Thyroid Association
EMT: Mesenchymal transition
DDR: DNA damage repair
CDF: Cumulative distribution function
SD: Standard deviation
LASSO: Least absolute shrinkage and selection operator
ROC: Receiver operating characteristic
AUC: Area under the ROC curve
HR: Hazard ratio
CI: Confidence interval
PSM: Propensity score matching
DFS: Disease-free survival
MFS: Metastasis-free survival
SNP: Single nucleotide polymorphism
C-index: Concordance index
