# Supplementary figures and images for: Novel molecular typing reveals the risk of recurrence in patients with early-stage papillary thyroid cancer
Source: Thyroid Res. 2024 Apr 1;17:7. doi: 10.1186/s13044-024-00193-9 (PMC10983671; doi:10.1186/s13044-024-00193-9)

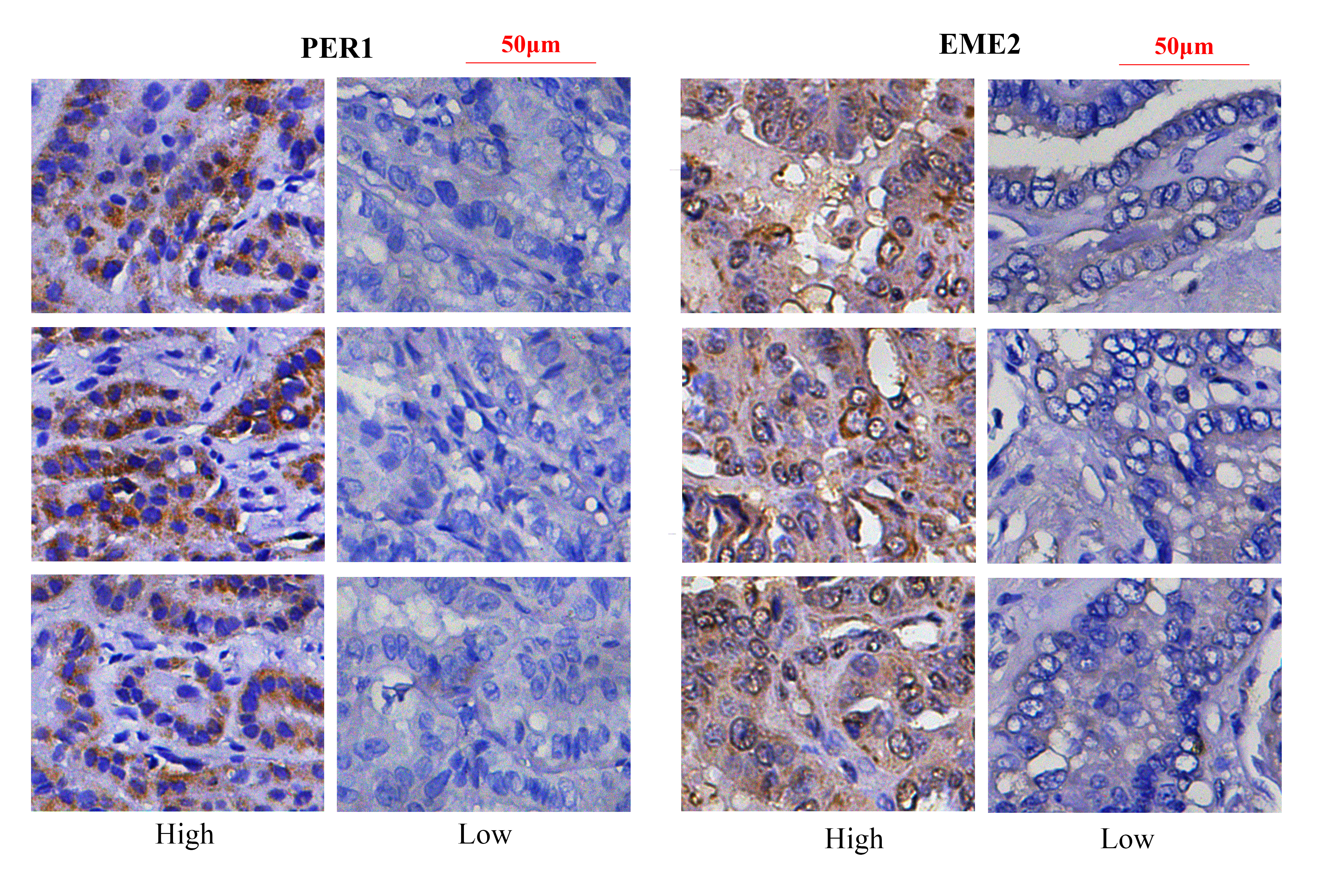

Supplement: Supplementary file 1 — Supplementary Fig.1. Representative images of IHC [file 13044_2024_193_MOESM1_ESM.jpg]
